# Analgesic Effects of Preincision Ketamine on Postspinal Caesarean Delivery in Uganda's Tertiary Hospital: A Randomized Clinical Trial

**DOI:** 10.1155/2017/5627062

**Published:** 2017-02-21

**Authors:** Richard Mwase, Tonny Stone Luggya, John Mark Kasumba, Humphrey Wanzira, Andrew Kintu, Joesph V. B. Tindimwebwa, Daniel Obua

**Affiliations:** ^1^Uganda People's Defense Forces Directorate of Medical Services, Bombo Military Hospital, Bombo, Uganda; ^2^Department of Anesthesia, College of Health Sciences, Makerere University, Kampala, Uganda; ^3^Directorate of Surgical Services, Mulago Hospital, Kampala, Uganda; ^4^Ministry of Health, Kampala, Uganda

## Abstract

*Background*. Good postoperative analgesic management improves maternal satisfaction and care of the neonate. Postoperative pain management is a challenge in Mulago Hospital, yet ketamine is accessible and has proven benefit. We determined ketamine's postoperative analgesic effects.* Materials and Methods*. We did an RCT among consenting parturients that were randomized to receive either intravenous ketamine (0.25 mg/kg) or placebo after spinal anesthetic. Pain was assessed every 30 mins up to 24 hours postoperatively using the numerical rating scale. The first complaint of pain requiring treatment was noted as “time to first breakthrough pain.”* Results*. We screened 100 patients and recruited 88 that were randomized into two arms of 44 patients that received either ketamine or placebo. Ketamine group had 30-minute longer time to first breakthrough pain and lower 24-hour pain scores. Postoperative diclofenac consumption was lesser in the ketamine group compared to placebo and Kaplan-Meier graphs showed a higher probability of experiencing breakthrough pain earlier in the placebo group.* Conclusion*. Preincision intravenous ketamine (0.25 mg/kg) offered 30-minute prolongation to postoperative analgesia requirement with reduced 24-hour pain scores. We recommend larger studies to explore this benefit. This trial is registered with Pan African Clinical Trial Registry number PACTR201404000807178.

## 1. Introduction

Pain is a subjective and multidimensional experience often inadequately managed in medical practice [1], with a USA survey reporting a 50–70% likelihood of experiencing moderate to severe postoperative pain due to inadequate pain management [2]. Good postoperative pain control improves maternal satisfaction and baby care, decreases morbidity [3, 4], and is desired to increase mobility which reduces the incidence of deep vein thrombosis [5]. In a low resource setting like Uganda, the National Referral and Teaching Hospital, Mulago, (MNRTH) faces profound challenges, for example, overwhelming patient numbers and anecdotal clinical staff shortages with a nurse: patient ratio of 1 : 40 [6]. Only forty-five percent (45%) of anesthesia providers have full-time access to pethidine or morphine while twenty-one percent (21%) have no complete access to these drugs [7].

Ketamine is an N-methyl-D-aspartate (NMDA) receptor antagonist that produces analgesia by desensitization of sensitized NMDA receptors, thereby inhibiting pain transmission in the CNS [8, 9]. In subanesthetic doses, it has been shown to reduce first 24 hours analgesic requirements after surgery with decreased postoperative opioid requirements in elective Caesarean section patients [10, 11]. Also for chronic pain it is a “third line” treatment where conventional treatments have failed [12]. Ketamine is safe for use in late pregnancy and for neonates and studies done in Africa have shown that it reduced morphine requirement after delivery among parturients [13]. It was recently cleared for use in brain injury patients based on its stable hemodynamic profile and beneficial bronchodilating respiratory properties, which has led to increased clinical use especially in severe asthma treatment [14].

We hypothesized that nonsedating dose of 0.25 mg/kg of preincision ketamine would prolong the time to pain requiring treatment, based on ketamine's beneficial properties above and especially since studies showed its benefit as an adjuvant for postoperative analgesia in low-dose regimens ranges of 0.25–0.5 mg/kg [15]. We chose a randomized prospective controlled trial setting to study the twenty-four-hour (24-hour) postoperative analgesia effects when administered at preincision for elective Caesarean delivery performed under spinal anesthesia.

## 2. Materials and Methods

This study was approved by the Makerere University, School of Medicine Research and Ethics Committee (SOMREC), and was registered to the Pan African Clinical Trial Registry with identifier number PACTR201404000807178. This was a prospective, randomized, double-blind, placebo-controlled trial conducted from January to March 2014 at MNRTH, which is a 1500-bed capacity that receives on average 48,000 patients annually at its Accident and Emergency (A&E). The labor suit conducts 32,000 deliveries annually with Caesarean sections accounting for 15–20% [16].

Eligible patients due for elective Caesarean section under spinal anesthesia were taken through consent process and enrolled after getting written consent. Our inclusion criteria included parturients scheduled for elective Caesarean section with American Society of Anesthesiologists (ASA) physical statuses I and II. Exclusion criteria were patients with hypertension, hypersensitivity to ketamine or its preservatives, ASA class > II, psychiatric disease, those who received chronic opioid therapy, and also those who required conversion from spinal to general anesthesia.

Sample size was calculated based on a power of 80% and standard deviation of 25 from study by Menkiti et al. [17] who used a ketamine dose of 0.15 mg/kg and noticed lower postoperative pain scores in the ketamine group, with the minimum expected difference (*D*) between the two means (*μ*_1_ − *μ*_2_), at 120 minutes. This gave us a total patient estimate (*N*) of eighty-eight (88) patients with forty-four (44) in each arm of the study.

### 2.1. Randomization and Concealment

Block randomization with a block of 4 was used to randomly assign participants to receive either ketamine or placebo in equal numbers for the two groups. A computer program was used to generate the randomization sequence by an independent statistician.

The ketamine group received intravenous ketamine (0.25 mg/kg) and the placebo group received normal saline and blinding for both participants and study investigators was achieved by use of 10 mL syringes of similar appearance and consistency. The placebo was 10 mL of normal saline while ketamine was diluted to make 10 mL of 5 mg/mL solution. All syringes had 5 mg/mL colorless solution that catered for a lowest possible adult weight of 40 kg and a highest weight being 200 kg. 

*Concealment*. Concealment was achieved by making sure that each syringe was labelled according to sequence-generated codes earlier presented as a list of sequential random treatment codes. The labelled syringes were brought in an opaque carrier envelope to the operating theatre and handed to the principle investigator every morning in the presence of a theatre nurse and study investigator. At the theatre receiving area a syringe was retrieved with its sticker code number, similar to computer generated number sequence, becoming the patients study number and then patient was weighed before transportation to the operating theatre (OR).

### 2.2. Study Procedures

Patient was weighed in the OR and received standard spinal anesthetic 2 mL of 0.5% heavy bupivacaine with 8% dextrose and 20 mcg of fentanyl and then placed supine with a left lateral tilt to achieve a sensory block height of T6 before surgery. Oxygen at 3 L/min was administered by nasal prongs to all patients. Immediately before surgical incision, the study drugs were administered as per randomized allocation of 0.05 mL/kg of study-drug or placebo as described above, followed by prophylactic intravenous antibiotics. Postoperative pain was assessed using the self-reporting numeric rating scale (NRS) with a rating of the lowest 0 = no pain, 10 = worst pain, 1–3 = mild pain, 4–6 = moderate pain, and 7–10 = severe pain. This was monitored every 30 mins for 24 hours or until first complaint of pain called the “time to first breakthrough pain” which was considered to be the pain requiring treatment (NRS ≥ 3). At this point rescue analgesic was administered firstly by intramuscular diclofenac (75 mg), then standard of care treatment, and then tramadol as secondary or meperidine/pethidine (100 mg) as tertiary for persistent pain.

### 2.3. Data Management

Interviewer-administered and pretested questionnaires were used for data collection. The data was cleaned, coded, and double-entered into Epidata version 3.1 and then exported and analyzed with STATA® Version 12 (Statacorp LP). The participants characteristics were presented as means and accompanying standard deviation for weight (kg), height (cm), and duration of surgery (in minutes) while median with range was estimated for age (in years) and parity as these were discreet variables. The differences in median (range) between those randomized to receive ketamine and those on placebo were assessed using a Wilcoxon rank sum test, while a *t*-test was used to test for the mean differences among the continuous variables. Survival analysis using the Kaplan-Meier survival curves was used to determine the probability of breakthrough pain between the two randomization arms with accompanying log-rank test to estimate the *p* value. In analysis, a *p* value of ≤ 0.05 was considered statistically significant.

## 3. Results

### 3.1. Baseline Characteristics

**Figure 1:**
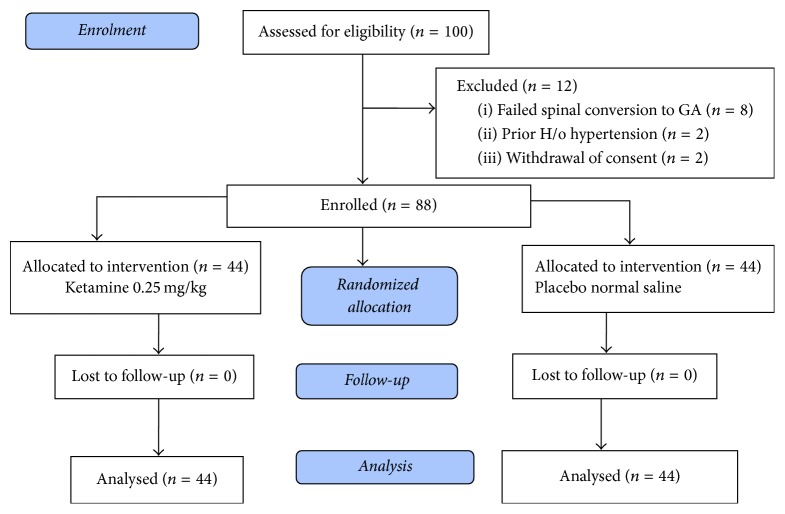
Flow chart and numerical scale.

**Figure 2:**
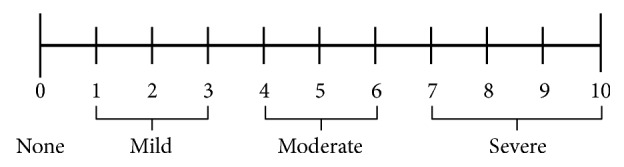
Numeric rating scale (NRS).

We screened 100 participants for this study and 12 were excluded while 88 parturients were enrolled with 44 assigned to one of the two groups (Figure 1). Overall, the median (range) age was 29 (14–44) years, with a median (range) parity of 4 (1–9) as shown in Table 1. The average (standard deviation) weight and height of enrolled mothers were 67 kg (10.91) and 160 cm (5.85), respectively. On average each operation took 40 minutes (12.75) with baseline maternal demographics equally distributed in both arms. NRS scale (Figure 2) was used to assess patients pain.

### 3.2. Breakthrough Pain

**Figure 3:**
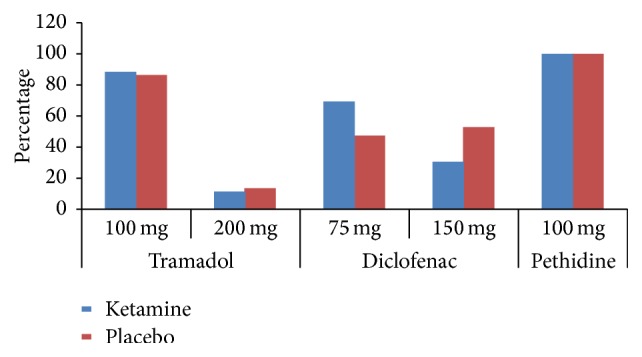
Graph showing postoperative analgesic drugs.

The median (range) time (in minutes) to first “breakthrough pain” among both groups was significantly longer in the ketamine group [210 (90–270)] when compared to those that received placebo [180 (90–360)] with *p* = 0.002. The 24-hour median (range) NRS pain scores were higher and significant in the placebo group [5 (3–7)] compared to the ketamine group [7 (3–9)] (*p* = 0.001) (Table 2). Primary rescue analgesia dose of diclofenac was lower in the ketamine group [used up to 75 mg] in comparison to the placebo [used up to 150 mg] (*p* value = 0.053). Secondary rescue analgesia with tramadol was equaled in both arms and therefore not statistically significant as shown in Figure 2 and Table 2. Eight patients (5-placebo and 3-ketamine) required a tertiary dose of pethidine (100 mg) due to persistent pain (Figure 3). At delivery all neonates scored APGAR 10 at 5 and 10 minutes while at study termination all mothers and newborns were in good general condition with stable vitals.

#### 3.2.1. Kaplan-Meier Survival Graph

**Figure 4:**
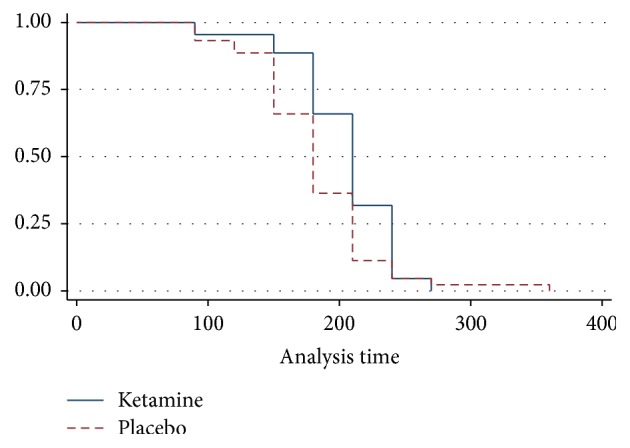
Kaplan-Meier survival curve. Ketamine versus placebo Kaplan-Meier survival estimates for breakthrough pain.

The probability of experiencing earlier breakthrough pain is significantly higher among those receiving placebo as compared to those who received ketamine with a log-rank test *p* = 0.011 (Figure 4). This showed ketamine significantly delayed the onset of breakthrough pain as compared to those who received placebo.

## 4. Discussion

We set out to determine the effects of low-dose ketamine on postoperative course when given intravenously at preincision after spinal anesthetic for Caesarean section delivery. Our study showed that preincision IV ketamine 0.25 mg/kg prolonged first requirement of pain treatment by 30 minutes with our ketamine group showing lower pain scores at 24 hours and also, albeit not statistically significant, had reduced cumulative analgesic administration. This showed promise for ketamine's postoperative clinical benefit especially in our resource limited setting. The first day after Caesarean delivery is crucial for maternal-child bonding and early initiation of breast feeding and a good long postoperative pain window allows clinicians to start other analgesic drugs before the anticipated onset of postoperative pain [17]. Our study findings are similar to clinical setting studies on ketamine that showed 10–30-minute prolongation breakthrough pain after outpatient knee arthroscopy [18].

Ketamine was chosen because it is a readily available drug in our setting and its multiple beneficial properties especially its NMDA blockade aid in postoperative analgesia based on the hypothesis that preemptive analgesia prevents induction of central sensitization by pain inputs [19]. Surgical tissue damage causes central pain pathway sensitization by glutamate release that is manifested clinically as heightened pain sensation, due to activation of postsynaptic NMDA receptors in the spinal cord [20]. Also NMDA receptors found both pre- and postsynaptically at pain fiber terminations in the dorsal horn contribute to “wind-up,” central sensitization plus long-term potentiation (LTP), all of which may contribute to initiation of abnormal pain; hence NMDA antagonists may prevent induction of central sensitization and diminish hypersensitivity [19]. Studies from West Indies showed ketamine reduced postoperative opioid requirement with coupled improved patient satisfaction [21]; however our study did not capture this as our local postoperative standard of care at study time was diclofenac followed by tramadol secondarily and then pethidine/meperidine as tertiary options for “persistent” pain. Ketamine in subanesthetic doses offered persistent analgesia on awakening because of its two main active metabolites and its elimination half-life of 180 minutes with analgesia [22]; we therefore postulated that ketamine be used for analgesia since studies have shown, other than the intravenous route, its efficacy after intramuscular and oral administration [23, 24]. Also its formulation is stable at room temperature with no need of special storage conditions and produces general anaesthesia within 30–60 seconds that lasts 10 to 15 minutes with ability to use repeated 1–3 mg/min boluses for maintenance which has made it easy to use in emergency prehospital care [25].

This obstetrics study was a pioneer ketamine RCT in MNRTH to show ketamine's potential as a postoperative adjunct to analgesia and was undertaken because globally postoperative pain remains an issue even in first world systems despite wide knowledge, new analgesics, and delivery techniques [2].

Our findings of 30-minute extension to time to breakthrough pain could in part be because we considered the lower threshold cutoff for pain as 3 on numerical scale unlike similar studies that used a higher threshold at the moderate pain level (4 for NRS and <2 for VAS) as a point of intervention [18], or because we gave it at preincision rather than at end of surgery hence observing a “shortened” time to next analgesic. We thus recommend more ketamine studies addressing and incorporating our perceived shortcomings that affected this study's time variable. However clinically in this study, ketamine was tolerated well among women and neonates with insignificant adverse effects and had similar outcomes to other studies [21].

### 4.1. Study Limitations

Limitations of this study are the following: low powered study unable to give statistically significant finings, inability to draw a solid conclusion for maternal and neonatal safety without monitoring ketamine serum levels as liquid chromatography- (LC-) tandem mass spectrometry for its metabolites were not available and would have been a steep cost for self-funded research, and failure to capture ketamine's long-term effects, preferably in postnatal clinic at 6 weeks as per MNRTH protocol, because we premised it on the subanesthetic dose used and study terminated at 48 hours postoperatively.

## 5. Conclusion

For Caesarean delivery Mulago, preincision intravenous ketamine (0.25 mg/kg) given after spinal anesthetic had a good safety profile and gave 30-minute prolongation to postoperative analgesia requirement with reduced 24 hr pain scores and findings are generalizable as biological interventions are usually similar across populations. However we recommend larger studies to further explore ketamine benefits and get the right dose estimate for prolongation of postoperative pain.

## Figures and Tables

**Table 1:** Patients' demographics.

Variable	Overall	Randomization group		*p* value
		Ketamine	Placebo	
Median age in years (range)	29 (14–44)	28 (14–44)	29 (18–41)	0.76
Median parity (range)	4 (1–9)	4 (1–9)	4 (1–7)	0.55
Mean weight in Kg (SD)	67 (10.91)	67 (10.96)	67 (10.97)	0.78
Mean height in cm (SD)	160 (5.85)	160 (5.52)	160 (6.22)	0.94
Mean duration of operation in minutes (SD)	40 (12.73)	40 (13.51)	39 (12.03)	0.72

**Table 2:** Showing primary and secondary outcomes.

Outcome	Randomization group		*p* value
	Ketamine Median (range)	Placebo Median (range)	
Time to breakthrough pain (minutes)	210 (90–270)	180 (90–360)	0.002
NRS at first breakthrough pain	5 (3–7)	7 (3–9)	0.001
Diclofenac used (mg)	75 (75–150)	150 (75–150)	0.05
Tramadol used (mg)	100 (100–200)	100 (100–200)	0.75

## Abbreviations

MNRTH: Mulago National Referral and Teaching Hospital
NMDA: N-Methyl-D-aspartate
ASA: American Society of Anesthesiologists
NRS: Numeric rating scale.
